# Taking a Load Off: User Perceptions of Smart Offloading Walkers for Diabetic Foot Ulcers Using the Technology Acceptance Model

**DOI:** 10.3390/s23052768

**Published:** 2023-03-02

**Authors:** M. G. Finco, Gozde Cay, Myeounggon Lee, Jason Garcia, Elia Salazar, Tze-Woei Tan, David G. Armstrong, Bijan Najafi

**Affiliations:** 1 Interdisciplinary Consortium for Advanced Motion Performance (iCAMP), Michael E. DeBakey Department of Surgery, Baylor College of Medicine, Houston, TX 77030, USA; 2 Keck School of Medicine, University of Southern California, Los Angeles, CA 90033, USA

**Keywords:** diabetic foot, smart offloading, remote patient monitoring, adherence, foot care, foot ulcer, wearable, digital health, telehealth

## Abstract

People with diabetic foot ulcers (DFUs) are commonly prescribed offloading walkers, but inadequate adherence to prescribed use can be a barrier to ulcer healing. This study examined user perspectives of offloading walkers to provide insight on ways to help promote adherence. Participants were randomized to wear: (1) irremovable, (2) removable, or (3) smart removable walkers (smart boot) that provided feedback on adherence and daily walking. Participants completed a 15-item questionnaire based on the Technology Acceptance Model (TAM). Spearman correlations assessed associations between TAM ratings with participant characteristics. Chi-squared tests compared TAM ratings between ethnicities, as well as 12-month retrospective fall status. A total of 21 adults with DFU (age 61.5 ± 11.8 years) participated. Smart boot users reported that learning how to use the boot was easy (ρ =−0.82, *p* ≤ 0.001). Regardless of group, people who identified as Hispanic or Latino, compared to those who did not, reported they liked using the smart boot (*p* = 0.05) and would use it in the future (*p* = 0.04). Non-fallers, compared to fallers, reported the design of the smart boot made them want to wear it longer (*p* = 0.04) and it was easy to take on and off (*p* = 0.04). Our findings can help inform considerations for patient education and design of offloading walkers for DFUs.

## 1. Introduction

Of the estimated 30 million people in the US with diabetes, 34% will develop a diabetic foot ulcer (DFU) in their lifetime [1]. DFUs, which precede 80% of amputation in people with diabetes, are associated with impaired physical function, reduced quality of life, and increased risk of death [2]. Ulcers requiring acute care can result in treatment costs of up to USD 70,000 per event, varying with the severity of the wound [3]. The annual direct cost related to DFUs in the US is almost USD 40 million, despite being a preventable complication of diabetes [4,5]. The standard of care for DFU management is protective offloading with either an irremovable or removable offloading boot, which allows the wound to heal while the person remains ambulatory [6,7,8,9].

However, inadequate adherence to the prescribed use of offloading devices could be a potential barrier to ulcer healing. Irremovable knee-high offloading devices are recommended for offloading intervention [10]. Removable offloading devices are recommended as a second option but are often more frequently prescribed than irremovable devices due to cost and healthcare team expectations of increased patient adherence [11]. People who wore offloading devices for 90 days had significantly higher acceptance of removable boots, compared to irremovable walkers or contact casts [12]. Despite higher rates of healing in irremovable devices [13,14], time to healing and amputation rates in removable walkers were comparable to the irremovable device literature [15]. Further, people who used removable walkers showed significantly more activity beginning at week 4, suggesting changes in adherence [15].

Despite adherence being a barrier to ulcer healing, few studies have investigated patient perceptions of offloading devices to help inform ways to improve adherence [16]. Several factors have been associated with low adherence to removable walkers, such as being male, a longer time with diabetes, not having peripheral arterial disease and higher perceived walker heaviness, as well as low wound healing and postural instability [17,18]. Additionally, a thematic analysis of people who wore a removable walker for anywhere between 1 week to 3 years found that although people reported they understood the benefits of the device, they also felt pressure from managers/coworkers not to wear it at work, did not like the height imbalance, and stated that the device felt heavy [19]. While studies have examined perceptions of offloading devices, no literature has examined perceptions surrounding the addition of technology offloading devices.

With advances in wearables, digital health, and remote patient monitoring technology, new solutions have emerged to help actively engage patients in caring for their wound, rather being passive recipients of wound care. However, patients’ acceptance of these solutions, as well as factors that may influence perceptions, are still unknown. Park et al. proposed the concept of smart offloading to reinforce adherence in using offloading devices and tested its proof of concept validity, comfort level, and ease of use in healthy adults without DFUs [20]. Further, Najafi et al. proposed the concept of smart insoles in people with a history of DFU and found users who received one alert every two hours were significantly more adherent to use their prescribed footwear [21]. To our knowledge, no prospective study has examined the acceptability and factors affecting adherence to smart-offloading devices for people with active DFUs. Thus, additional literature on perspectives of offloading devices with and without technology could help inform ways to promote adherence in people with DFUs. This knowledge could help inform factors that may be associated with the acceptance of smart offloading, particularly in older adults with diabetic foot syndrome.

This study is the first to explore perceptions surrounding smart offloading with real-time feedback in people with DFUs, and what participant characteristics may be associated with acceptability. The objective of this study was to examine user perspectives of irremovable, removable, and sensorized offloading walkers to provide insight on ways to help promote adherence. This study also sought to gain insight about factors associated with the acceptance of a smart offloading device with a remote patient monitoring component. Research outcomes were user perspectives on offloading boots, which were expressed through a questionnaire based on the Technology Acceptance Model.

## 2. Materials and Methods

This manuscript presents preliminary qualitative findings from an ongoing parallel randomized control trial (ClinicalTrials.gov Identifier: NCT04460573) to investigate the influence of a sensorized offloading walker on health outcomes in people with DFUs, termed Smart Monitoring of patient Activity via Remote Technologies for Best Optimizing Offloading Therapy (SMARTBOOT). All participants signed an approved consent form before enrolling in this study. The study protocol and consent form were approved by the University of Southern California Institutional Review Board (protocol number: HS-20-00526). A computer-generated list (MATLAB software) randomly assigned participants in a 1:1:1 ratio to one of three offloading device groups: (1) irremovable cast walker (iRCW, reference group), (2) original removable cast walker (oRCW, control group), or (3) smart removable cast walker (sRCW, intervention group). The offloading component was identical between groups and only the method for managing adherence was different. All participants wore their offloading device for 12 weeks, or until their ulcer was deemed healed by a physician. 

**Figure 1:**
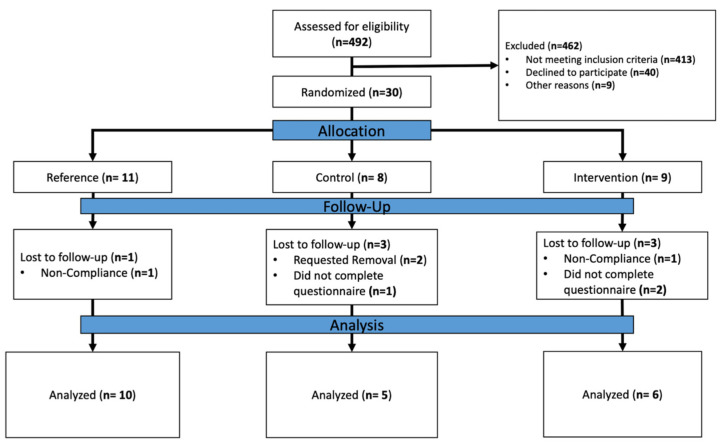
Consolidated Standards of Reporting Trials (CONSORT) diagram for inclusion and exclusion of participants.

Participants were recruited from the Keck School of Medicine (Los Angeles Metropolitan, CA, USA). To be included in the study, individuals had to be over 18 years of age, have diabetes mellitus, have a plantar ulcer, have evidence of peripheral neuropathy, and be ambulatory at home with or without assistance, and be willing and able to provide informed consent. Individuals were excluded from participating in the study if they had major foot deformity so that the patient could not fit to standard offloading (e.g., Charcot neuroarthropathy), active infection, major lower limb amputation, changes in psychotropic or sleep medication in the last 6 weeks, any clinically significant medical or psychiatric condition, severe cognitive impairment, or laboratory abnormality that would interfere with the ability to participate in the study. Additionally, individuals were excluded from participating in the study if they were being considered for revascularization during the study, concurrently participating in exercise training, or unable or unwilling to attend prescribed clinic visits or comply with protocol. Only participants who completed all self-report data (TAM and all participant characteristics reported in this manuscript) were included in the analysis. Figure 1 depicts the number of participants assessed for eligibility, and those who were excluded or included. After providing informed consent, demographics were collected, which included age, sex, weight, height, and number of 12-month self-reported retrospective falls. 

**Figure 2:**
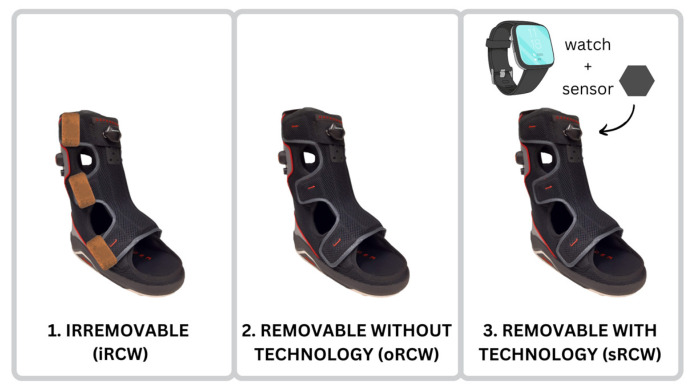
Participants were randomized to wear (1) an irremovable cast walker (iRCW), (2) an original removable cast walker that is standard of care (oRCW; OG indicates original gadget), or (3) a smart removable cast walker designed to provide feedback on adherence via a sensor and smartwatch (sRCW).

Study groups are depicted in Figure 2. The iRCW was sealed with patches of leather, so participants could not readily remove the boot, the oRCW was off-the-shelf with no additional modifications, and the sRCW had a sensor-based system that was designed to provide feedback on adherence.

**Figure 3:**
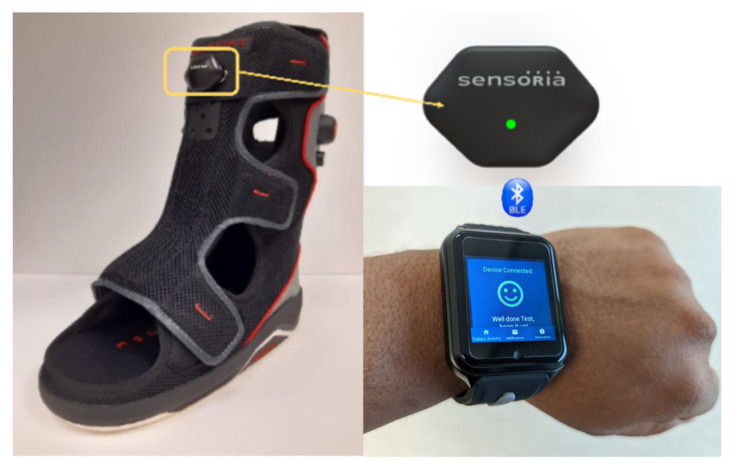
The overall smart offloading system, used by participants assigned to use the smart removable cast walker (sRCW). The system consists of a sensor that attaches to the cast walker, as well as a watch that provides the participant notifications regarding their adherence.

The sRCW and its validity were described in detail in our prior publication on healthy controls [20]. In summary, sRCW includes an identical offloading as iRCW and oRCW, however it uses a six-degree-of-freedom inertial measurement unit (Sensoria Health, Seattle, WA, USA, Figure 3) attached on the strut of offloading, enabling real-time measurement of adherence, walking steps, and walking cadence. Participants received real-time feedback about adherence and walking steps using a smartwatch with a dedicated patient monitoring app. The Bluetooth Low Energy module enabled real-time communicate of parameters of interest with the smartwatch. The microcontroller in the smartwatch processed the data and showed real-time (with maximum 5 s lag time) boot condition (boot on or boot off), activity condition (active or resting), step count, and notifications. Additionally, data were streamed to a secured cloud-based system for a remote patient monitoring solution, via a 4G LTE Internet of Things (sim card enabled). This allowed the remote monitoring of parameters of interest (e.g., adherence, daily steps with and without adherence, and cadence), which could be used by clinicians to personalize patient education during weekly visits. Participants received real-time notifications from their smartwatch to encourage adherence via visual (e.g., happy face for good adherence, sad face for poor adherence) and vibration/audio feedback (walking while not wearing offloading). Additionally, participants had a daily comprehensive report via watch interface about level of adherence and daily steps. 

Participants completed the following patient-reported outcomes: the Montreal Cognitive Assessment (MoCA) to assess cognition [22], the Falls Efficacy Scale International (FES-I) to assess falls efficacy [23], and the Patient Reported Outcomes Measurement Information System (PROMIS-29) to assess quality of life [24]. 

**Figure 4:**
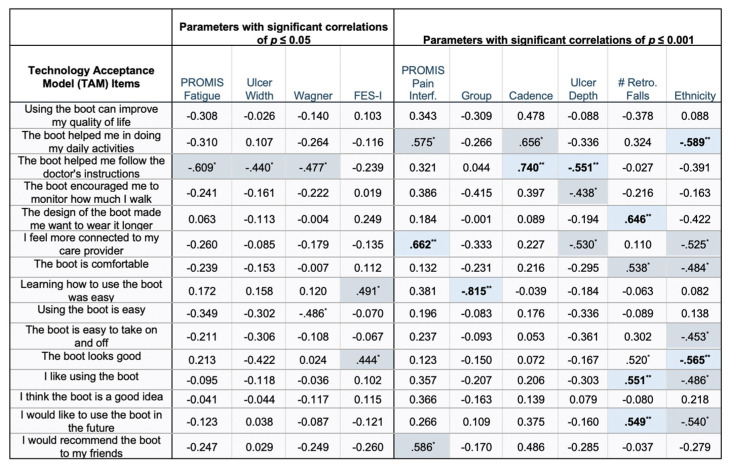
Significant Spearman correlations between participant ratings on Technology Acceptance Model (TAM) Questionnaire items and participant characteristics. Ratings were coded as 1= strongly agree, 2 = agree, 3 = neutral, 4 = disagree, and 5 = strongly disagree. Significance was considered *p* ≤ 0.05. *p*-values with asterisks (*) and dark blue shading denotes significance of *p* ≤ 0.05. Bold text with two asterisks (**) and light blue shading denotes significance of *p* ≤ 0.001, which are discussed in the main text. Non-significant correlations are listed in Supplementary Table S1. Abbreviations: MoCA = Montreal Cognitive Assessment, PROMIS = Patient-Reported Outcome Measurement Information System, FES-I = Falls Efficacy Scale International.

To assess perspectives on device acceptability, participants also completed a 15-item questionnaire based on the Technology Acceptance Model (TAM) [25], with a 5-point Likert scale, with the following options: strongly disagree, disagree, neutral, agree, and strongly agree. The 15 items are listed in Figure 4. 

Participants who reported identifying as Hispanic or Latino were classified as Hispanic or Latino, while those who did not were classified as Non-Hispanic or Latino. Additionally, participants who reported experiencing at least one fall in the past 12 months were categorized as fallers, while those who did not report experiencing any falls in the past 12 months were categorized as non-fallers.

Spearman correlations were performed to determine associations between participant characteristics and TAM ratings. Chi-squared tests of independence were performed to examine the relationship between TAM ratings with ethnicity (Hispanic or Latino, Non-Hispanic or Latino), as well as TAM ratings with 12-month retrospective fall status (faller, non-faller). All statistical analyses were performed using IBM SPSS Statistics 25 (IBM, Chicago, IL, USA). Statistical significance in all tests was considered to be a 2-sided *p*-value of *p* ≤ 0.05.

## 3. Results

A total of 21 adults with DFUs (age 61.5 ± 11.8 years; 85.7% male) were randomized to use an iRCW (n = 10), oRCW (n = 6), or sRCW (n = 5). Participant characteristics by ethnicity and fall status are depicted in Table 1. People who identified as Hispanic or Latino, compared to those who did not, had significantly higher cadence (Table 1). Fallers, compared to non-fallers, had significantly higher T-scores on PROMIS-Cognitive Function and PROMIS-Depression items, which is interpreted as having higher indications of cognitive function and depression (Table 1).

The majority of participant characteristics had no significant correlations with any of their self-reported TAM ratings, which are presented in Supplementary Table S1. Correlations with significance are depicted in Figure 4. Due to the high number of correlations with a *p*-value of *p* ≤ 0.05, only those with a *p*-value of *p* ≤ 0.001 are discussed. Participants who used the smart boot (ρ = −0.82, *p* < 0.001) reported that learning how to use the boot was easy (Figure 4). Participants who had lower cadence (ρ = 0.74, *p* < 0.001) or deeper ulcers (ρ = −0.55, *p* < 0.001) reported that the boot helped them follow physician orders (Figure 4). Participants with lower T-scores on the PROMIS-Pain Interference, indicating less pain, reported feeling more connected to their care provider (ρ = 0.66, *p* < 0.001) (Figure 4).

Chi-squared results by ethnicity and fall status are depicted in Table 2. Individuals who identified as Hispanic or Latino reported the boot helped with their daily activities (ρ = −0.59, *p* < 0.001) and looked good (ρ = −0.57, *p* < 0.001). Individuals who identified as Hispanic or Latino, compared to those who did not, reported they liked using the boot (*p* = 0.05) and would like to use it in the future (*p* = 0.04). Individuals with fewer retrospective falls reported the boot’s design made them want to wear it longer (ρ = 0.65), they liked using it (ρ = 0.55), and would like to use it more in the future (ρ = *0*.55) (all *p* < 0.001). Non-fallers, compared to fallers, reported the design of the boot made them want to wear it longer (*p* = 0.04) and it was easy to take on and off (*p* = 0.04). 

## 4. Discussion

This study sought to examine user perspectives of irremovable, removable, and sensorized offloading boots (smart boot) to provide insight on ways to help promote adherence and gain insight about factors associated with the acceptance of a smart offloading device with a remote patient monitoring component. Correlation results suggest smart offloading may ultimately help promote adherence, since sensorized boot users were more inclined to report that learning how to use the boot was easy. Additionally, participants with lower cadence or deeper ulcers tended to report that the boot helped them follow physician instructions, regardless of group. Chi-squared results suggest that participants who identified as Hispanic or Latino, as well as those who had fewer or no retrospective falls, tended to rate their offloading boot more favorably regardless of group. These findings provide supporting evidence that older adults could find a sensorized offloading boot easy to use for DFU management. Further, people who do not identify as Hispanic or Latino, report falling in the past 12 months, or report less severe symptoms (e.g., higher cadence, shallower ulcer) may need additional targeted patient education to promote adherence. 

Age, dropout from the study, group assignment, fear of falling, or cognition did not show significant associations with TAM ratings. Based on previous work that has indicated people prefer lower-profile walkers that are removable [12], we expected TAM ratings would differ by group assignment. However, participants only wore and rated the one walker they were assigned. Future research could examine preferences after using multiple walker types. Additionally, we expected cognition and age would be associated with perceptions on ease of use and the adoption of technology (e.g., impaired eyesight, dexterity, ability self-care) [26,27,28]. Future work could focus on examining if age or cognition influences perceptions of sensorized boot use or adherence.

In previous research, people with diabetes who identify as Hispanic or Latino have been shown to experience higher rates of foot ulcers and subsequent amputations, be more likely to develop chronic foot wounds despite receiving regular care, and be less likely to receive diabetic foot care and attempted limb salvage [29,30,31,32]. In this study, findings indicated that people who identified as Hispanic or Latino tended to report the offloading boots more favorably, regardless of group. This suggests the overall design of the boot, regardless of the modifications to the boot in each of the three groups, may help reduce ethnicity-related health disparities in DFU management. Higher cadence may also influence more favorable perceptions, since people who identified as Hispanic or Latino also had significantly higher cadence compared to those who did not identify as Hispanic or Latino. To help determine this, future work could examine open-ended perceptions of participants to determine what aspects of the boot they thought “looked good” or “helped with their daily activities” when rating those items favorably.

Participants who reported having fewer falls in the past 12 months (correlation results) or were non-fallers (Chi-squared results) tended to report more favorable perceptions, particularly regarding the design and ease of taking the device on and off. This appears to be aligned with prior work that found postural instability was a factor associated with low adherence to boot use [18]. However, no significant relationship of *p* < 0.001 was found between fear of falling and TAM ratings. This suggests that self-reported number of 12-month falls may be a better indicator of boot acceptability than fear of falling. Fallers also had significantly higher indications of cognitive function (better) and depression (worse) compared to non-fallers, which may have also influenced perceptions. More work is needed to directly examine these relationships.

This study had a limited sample size of 21 participants, and acceptability was determined by a single questionnaire. Our findings could help inform directions for a thematic analysis, which would provide more detailed user perceptions on specific factors to help promote adherence. While this study focused on patient factors, the WHO recommends four other dimensions of factors (social/economic, therapy-related, condition-related, and health-system related) that should also be considered [33]. For example, participant hygiene or exposure to physical therapy could also influence acceptability.

## 5. Conclusions

Overall, findings from this study suggest that smart offloading with a remote patient monitoring solution may help promote adherence among older adults to wear offloading boots prescribed for DFUs. The design of the particular walker that was used in this study, regardless of being irremovable or removable, was better accepted among people who identified as Hispanic or Latino. Further, findings suggest clinicians could provide additional patient education for people who report experiencing at least one fall over the previous 12 months, particularly in putting on and taking off the walker. Manufacturers could also consider designs that improve perceptions of stability and appearance of the walker. Ultimately, smart technology and considerations surrounding ethnicity and fall status may help improve adherence in older adults with DFUs who are prescribed offloading walkers.

## Figures and Tables

**Table 1:** Participant Characteristics by Ethnicity and Fall Status.

	Hispanic or Latino (n = 14)	Non-Hispanic or Latino (n = 7)	*p*-Value	Fallers (n = 8)	Non-Fallers (n = 13)	*p*-Value
Randomized Group	50% iRCW, 21.4% oRCW, 28.6% sRCW	42.9% iRCW, 14.3% oRCW, 14.3% sRCW	0.675	12.5% iRCW, 50% oRCW, 37.5% sRCW	69.2% iRCW, 7.1% oRCW, 23.1% sRCW	0.057
Age (years)	60.7 ± 13.3	60.1 ± 12.7	0.693	59.2 ± 16.8	60.5 ± 10.3	0.901
BMI (kg/m^2^)	27.3 ± 5.4	58.9 ± 17.7	0.155	32.2 ± 14.8	29.0 ± 5.7	0.570
Ethnicity (% Hispanic or Latino)	100	0	0.001 *	100	0	0.252
Sex (% Male)	92.9	85.7	0.400	90.5%	87.5%	0.001 *
#12-month retrospective falls	0.4 ± 0.9	2.3 ± 3.1	0.200	1.1 ± 2.0	0.0 ± 0.0	0.001 *
Healing time (weeks)	7.1 ± 4.5	7.5 ± 4.2	0.482	7.2 ± 4.2	5.9 ± 4.3	0.770
Cadence (steps/min)	66.8 ± 30.0	47.8± 43.5	0.015 *	62.7 ± 32.4	68.0 ± 38.3	0.121
HbA1C (%)	6.7 ± 3.1	8.0 ± 0.9	0.194	7.1 ± 2.7	6.5 ± 3.0	0.095
MoCA Score	10.5 ± 5.9	12.0 ± 8.8	0.610	11.0 ± 6.8	10.3 ± 5.9	0.601
FES-I Score	24.5 ± 18.5	31.4 ± 24.4	0.295	26.9 ± 20.4	27.0 ± 20.1	0.288
Wagner Score	1.7 ± 1.2	1.6 ± 0.8	0.833	1.7 ± 1.1	1.6 ± 1.2	0.771
Ulcer Characteristics						
Length (cm)	1.4 ± 1.5	1.1 ± 0.4	0.357	1.3 ± 1.2	1.4 ± 1.5	0.560
Depth (cm)	0.2 ± 0.3	0.1 ± 0.1	0.078	0.8 ± 0.2	0.2 ± 0.3	0.933
Width (cm)	1.8 ± 1.8	1.0 ± 0.7	0.154	1.6 ± 1.5	1.7 ± 1.9	0.459
Area (cm^2^)	3.1 ± 5.6	1.0 ± 0.9	0.174	2.4 ± 4.7	3.1 ± 5.8	0.295
PROMIS T-Scores						
Pain Interference	53.5 ± 13.9	58.6 ± 10.1	0.221	55.4 ± 12.6	55.2 ± 13.5	0.805
Cognitive Function	34.2 ± 5.8	36.1 ± 7.5	0.568	34.9 ± 6.3	32.5 ± 4.8	0.035 *
Depression	47.3 ± 7.4	51.0 ± 11.1	0.220	48.6 ± 8.9	45.4 ± 7.2	0.039 *
Social Function	48.2 ± 13.1	47.8 ± 16.0	0.373	48.1 ± 13.8	50.1 ± 13.4	0.245
Anxiety/Fear	47.7 ± 10.5	47.0 ± 11.4	0.869	47.5 ± 10.5	46.0 ± 10.2	0.312
Fatigue	42.9 ± 14.3	48.6 ± 12.3	0.168	45.0 ± 13.5	43.3 ± 13.9	0.556
Physical Function	32.9 ± 10.8	31.9 ± 9.1	0.200	32.5 ± 10.0	32.5 ± 11.1	0.766
Sleep Disturbance	54.5 ± 3.1	50.7 ± 5.2	0.223	53.1 ± 4.3	53.5 ± 2.8	0.578

Table 1: Participant characteristics by ethnicity (Hispanic or Latino, Non-Hispanic or Latino) and fall status (fallers, non-fallers). Kruskal–Wallis (randomized study group), Chi-squared (ethnicity, sex) and Mann–Whitney U tests (all other items) were used to determine statistical significance. 2-sided *p*-values of *p* ≤ 0.05 were considered significant. Bolded *p*-values with asterisks (*) denote significance.

**Table 2:** Chi-Squared Results by Ethnicity and Fall Status.

TAM Questionnaire Items	Hispanic or Latino (n = 13) SA/A/N/D/SD	Non-Hispanic or Latino (n = 8) SA/A/N/D/SD	χ2	*p*-Value	Fallers (n= 8) SA/A/N/D/SD	Non-fallers (n = 13) SA/A/N/D/SD	χ2	*p*-Value
Using the boot can improve my quality of life	8/5/0/0/0	6/1/1/0/0	2.928	0.231	7/1/0/0/0	7/5/1/0/0	2.625	0.269
The boot helped me in doing my daily activities	7/4/1/1/0	1/1/2/1/3	8.950	0.062	3/0/1/1/3	5/5/2/1/0	8.102	0.088
The boot helped me follow the doctor’s instructions	9/2/2/0/0	2/4/1/1/0	5.580	0.134	4/3/0/1/0	7/3/3/0/0	3.846	0.279
The boot encouraged me to monitor how much I walk	6/3/4/0/0	2/3/3/0/0	1.010	0.604	4/3/1/0/0	4/3/6/0/0	2.524	0.283
The design of the boot made me want to wear it longer	2/5/3/2/1	1/0/2/2/3	5.664	0.226	0/1/1/2/4	3/4/4/2/0	9.975	0.041 *
I feel more connected to my care provider	6/4/3/0/0	1/1/4/2/0	6.704	0.082	3/1/2/2/0	4/4/5/0/0	4.281	0.233
The boot is comfortable	2/7/1/2/1	1/0/1/3/3	7.784	0.100	0/1/1/3/3	3/6/1/2/1	6.976	0.137
Learning how to use the boot was easy	7/2/4/0/0	5/1/2/0/0	0.151	0.927	5/2/1/0/0	7/1/5/0/0	2.272	0.321
Using the boot is easy	4/6/2/1/0	4/2/2/0/0	1.918	0.049 *	3/4/1/0/0	5/4/3/1/0	1.388	0.708
The boot is easy to take on and off	5/5/2/1/0	1/2/1/3/1	5.401	0.249	1/3/0/4/0	5/4/3/0/1	10.197	0.037 *
The boot looks good	1/6/5/0/1	0/0/5/1/2	7.572	0.109	0/1/4/0/3	1/5/6/1/0	7.289	0.121
I like using the boot	2/7/2/1/1	1/0/1/5/1	9.692	0.046 *	0/2/0/4/2	3/5/3/2/0	9.288	0.054
I think the boot is a good idea	9/3/1/0/0	7/1/0/0/0	1.123	0.570	6/2/0/0/0	10/2/1/0/0	0.858	0.651
I would like to use the boot in the future	4/5/2/2/0	1/1/0/2/4	9.834	0.043*	0/2/0/3/3	5/4/2/1/1	8.986	0.061
I would recommend the boot to my friends	7/4/2/0/0	2/4/1/0/1	3.096	0.377	4/2/1/0/1	5/6/2/0/0	2.389	0.496

Table 2: Rating counts of participants who selected strongly agree (SA)/agree (A)/neutral (N)/disagree (D)/strongly disagree (SD) on the Technology Acceptance Model (TAM) questionnaire items. Chi-squared tests of independence were performed to assess significant differences between ethnicity (Hispanic or Latino, Non-Hispanic or Latino) and fall status (fallers, non-fallers). Chi-squared values (χ2) are depicted. 2-sided *p*-values of *p* ≤ 0.05 were considered significant. Bolded *p*-values with asterisks (*) denote significance.

## Data Availability

Data are available upon request by contacting the corresponding author.

## Supplementary Materials

The following supporting information can be downloaded at: https://www.mdpi.com/article/10.3390/s23052768/s1, Table S1: Correlations between TAM ratings and participant characteristics that were non-significant.
